# Comparative Proteomic Analysis of Serum from Patients with Systemic Sclerosis and Sclerodermatous GVHD. Evidence of Defective Function of Factor H

**DOI:** 10.1371/journal.pone.0012162

**Published:** 2010-08-13

**Authors:** Cinzia Scambi, Valentina La Verde, Lucia De Franceschi, Giovanni Barausse, Fabio Poli, Fabio Benedetti, Marco Sorio, Francesca Deriu, Paola Roncada, Oscar Bortolami, Francesco Turrini, Paola Caramaschi, Chiara Stranieri, Lisa M. Bambara, Domenico Biasi

**Affiliations:** 1 Section of Rheumatology, Department of Clinical and Experimental Medicine, University of Verona, Verona, Italy; 2 Section of Internal Medicine, Department of Clinical and Experimental Medicine, University of Verona, Verona, Italy; 3 Section of Haematology, Department of Clinical and Experimental Medicine, University of Verona, Verona, Italy; 4 Department of Veterinary Pathology, Hygiene and Public Health (DIPAV), University of Milano, Milano, Italy; 5 Section of Proteomics, Istituto Sperimentale Italiano L. Spallanzani, University of Milano, Milano, Italy; 6 Unit of Epidemiology and Medical Statistics, Department of Medicine and Public Health, University of Verona, Verona, Italy; 7 Section of Medical Chemistry, Department of Genetic, Biological and Medical Chemistry, University of Torino, Torino, Italy; University of Toronto, Canada

## Abstract

**Background:**

Systemic sclerosis (SSc) is an autoimmune disease characterized by immunological and vascular abnormalities. Until now, the cause of SSc remains unclear. Sclerodermatous graft-versus-host disease (ScGVHD) is one of the most severe complications following bone marrow transplantation (BMT) for haematological disorders. Since the first cases, the similarity of ScGVHD to SSc has been reported. However, both diseases could have different etiopathogeneses. The objective of this study was to identify new serum biomarkers involved in SSc and ScGVHD.

**Methodology:**

Serum was obtained from patients with SSc and ScGVHD, patients without ScGVHD who received BMT for haematological disorders and healthy controls. Bi-dimensional electrophoresis (2D) was carried out to generate maps of serum proteins from patients and controls. The 2D maps underwent image analysis and differently expressed proteins were identified. Immuno-blot analysis and ELISA assay were used to validate the proteomic data. Hemolytic assay with sheep erythrocytes was performed to evaluate the capacity of Factor H (FH) to control complement activation on the cellular surface. FH binding to endothelial cells (ECs) was also analysed in order to assess possible dysfunctions of this protein.

**Principal Findings:**

Fourteen differentially expressed proteins were identified. We detected pneumococcal antibody cross-reacting with double stranded DNA in serum of all bone marrow transplanted patients with ScGVHD. We documented higher levels of FH in serum of SSc and ScGVHD patients compared healthy controls and increased sheep erythrocytes lysis after incubation with serum of diffuse SSc patients. In addition, we observed that FH binding to ECs was reduced when we used serum from these patients.

**Conclusions:**

The comparative proteomic analysis of serum from SSc and ScGVHD patients highlighted proteins involved in either promoting or maintaining an inflammatory state. We also found a defective function of Factor H, possibly associated with ECs damage.

## Introduction

*Systemic sclerosis* (SSc) is an autoimmune disorder characterised by microvascular endothelial cell (EC) apoptosis, excessive extracellular matrix protein deposition and perivascular infiltration of mononuclear cells in skin and affected organs. SSc is an heterogeneous disorder in terms of disease symptoms and clinical course, which has been classified into *limited SSc* (lSSc) and *diffuse SSc* (dSSc) [1], [2]. lSSc affects only the skin of distal extremities and face and is usually characterized by a very slow clinical course, whereas dSSc affects wide areas of skin and internal organs and may have severe pulmonary, cardiac, gastrointestinal and renal involvement. To date, no completely effective treatment is available for SSc, mainly related to the lack of knowledge of its pathogenesis. Increasing evidence suggests that several environmental events and a host-specific susceptibility may be important in the development of SSc [3]–[9]. An interesting model has been suggested for the pathogenesis of SSc, in which viral or bacterial infections and toxic agents lead to the production of auto-reactive cellular and humoral immune responses resulting in EC death and extracellular matrix protein deposition, in a genetically predisposed host [5], [9].

*Graft-versus-host disease* (GVHD) is an immunological disorder that occurs in approximately half of patients receiving allogenic bone marrow transplantation (BMT) for haematological disorders. It is usually classified as acute or chronic based on the time of onset and clinical manifestations. Acute GVHD usually occurs within 2 to 6 weeks following BMT and primarily affects the skin, the liver and the gastrointestinal tract. Chronic GVHD appears at least 2 or 3 months after allogenic BMT and may be progressive (acute GVHD merging into chronic), quiescent (acute GVHD that resolves completely but is later followed by chronic GVHD) or it may occur *de novo*. The manifestations of chronic GVHD are somewhat protean and often show similarities with autoimmune diseases. *Sclerodermatous GVHD* (ScGVHD) is a complication that appears in 10–15% of patients with chronic GVHD [10]. ScGVHD is characterized by clinical manifestations similar to SSc, including sclerotic skin. The etiologic factors of ScGVHD are still unclear and its classification as an autoimmune disorder has not yet been established. In fact, the presence in serum of ScGVHD patients of antibodies against cellular antigens is rare and non-specific, whereas they are present in almost all SSc patients [8], [11].

Here, we carried out a comparative proteomic analysis of serum from lSSc, dSSc, ScGVHD patients and control subjects to identify new biomarkers possibly involved in the pathogenesis of these disorders [12]. We found fourteen proteins differently expressed in patients compared to controls, which could play an important role in either promoting or maintaining a chronic inflammatory state in subjects affected by SSc or ScGHVD.

## Materials and Methods

### Ethics

The institutional ethics committee of Verona Hospital approved the experimental protocol. All subjects provided written informed consent before enrolment.

### Patient selection

We enrolled patients with lSSc (*n* = 11) and dSSc (*n* = 15), patients with ScGVHD *(n* = 8) and without ScGVHD (*n* = 5) who received BMT for haematological diseases and age- and sex-matched healthy subjects (*n* = 15). Patients referred to our Department for standard care and clinical evaluation, including serum determinations of anti-nuclear (ANA) and anti-extractable nuclear antigen (anti-ENA) antibodies, which had been determined according to standardised protocols.

### Blood collection

Venous blood was drawn from each subject into two 7 ml fasting blood tubes and allowed to clot at room temperature for 1 hour. Serum was separated by centrifugation at 2000×*g* for 15 min at 4°C, aliquoted and stored at −80°C.

When transplanted patients (T) were enrolled, blood samples were collected before BMT and at the moment of ScGVHD diagnosis. T patients without ScGVHD were evaluated at the same time of those who developed ScGVHD. Serum from SSc patients was used within one year, whereas serum from onco-haematologic patients was used within three years from the collection.

### Bidimensional electrophoresis (2D) analysis

Total serum proteins were determined by BCA Protein Assay Kit (Thermo Scientific, Rockford, IL., USA). Serum was diluted in buffer containing 8 M urea (Fluka, Buchs, Switzerland), 4% CHAPS (USB, Cleveland, OH, USA), 40 mM Tris (Sigma/Aldrich, St Louis, MO, USA), 1% Dithiothreitol (DTT, Fluka), 2% IPG-buffer (GE Healthcare, Little Chalfont, UK) and traces of bromophenol blue (Sigma/Aldrich). Immobiline dry strips (pH range 3–10, 4–5.5, GE Healthcare) were rehydrated overnight in Rehydratation Solution containing 8 M urea, 2% CHAPS, 1% DTT and 1.5% IPG buffer pH 4–7. For the isoelectrofocusing, 50 µl of the diluted serum sample at the final concentration of 12 µg/µl were loaded in two cups put at the positive and negative side of the IPG strip. Isoelectric focusing was performed using IPGphor electrophoresis unit (GE Healthcare). Total voltage applied was 55 KV. Subsequently, IPG strips were reduced in Equilibration buffer (0.5 mM Tris-HCl pH 6.8, 6 M urea, 30% glycerol (Sigma/Aldrich), 2% SDS (Sigma/Aldrich) containing 1% DTT. Strips were then alchilated in the same buffer containing 2.5% iodoacetamide (Fluka) instead of DTT.

The second dimension was run in a Hoefer 600 apparatus (Hoefer, Holliston, USA) and proteins were separated into a 9–16% polyacrilamide gels; gels were stained with colloidal Coomassie. Based on preliminary data on the reproducibility of the 2D maps, we generated 2D maps from serum of each patient and healthy control.

Then, gels underwent to image analysis by the image master 2D Platinum software (GE Healthcare). Spots were analyzed by statistic tests to select those statistically significantly expressed in the comparison between groups.

### Electrophoresis fractionation and in situ digestion

Selected protein spots were excised from 2D gels and washed in 50 mM ammonium bicarbonate pH 8.0 in 50% acetonitrile to a complete destaining. The gel pieces were re-suspended in 50 mM ammonium bicarbonate pH 8.0, containing trypsin 100 ng, incubated for 2 hrs at 4°C and overnight at 37°C. The supernatant containing the resulting peptide mixtures was removed and the gel pieces were re-extracted with acetonitrile. The two fractions were then collected and freeze-dried.

### MALDI MS analysis

MALDI mass spectra were recorded on an Applied Biosystem Voyager DE-PRO mass spectrometer equipped with a reflectron analyser and used in delayed extraction mode. 1 µl of peptide sample was mixed with an equal volume of α-cyano-4-hydroxycynnamic acid as matrix (10 mg/ml in 0.2% TFA in 70% acetonitrile), applied to the metallic sample plate and air dried. The acceleration voltage was 20 kV, delay time 100 ns and the grid voltage was set to 73%. Spectra were acquired from 600 to 5000 Da using 1000 laser shots/spectrum. Mass calibration was performed by using the standard mixture provided by the manufacturer. The typical error was in the 50–100 ppm range. Mass signals were then used for database searching using the MASCOT peptide fingerprinting search program (Matrix Science, Boston, USA) available on the net.

### Liquid chromatography-tandem mass spectrometry (LC-MS/MS) analysis

When the identity of the proteins could not be established by peptide mass fingerprinting, the peptide mixtures were further analyzed by LCMSMS using the LC/MSD Trap XCT Ultra (Agilent Technologies, Palo Alto, CA) equipped with a 1100 HPLC system and a chip cube (Agilent Technologies). After loading, the peptide mixture (7 µl in 0.5% TFA) was first concentrated and washed (i) at 1 µl/min onto a C18 reverse-phase pre-column (Waters) or (ii) at 4 µl/min in 40 nl enrichment column (Agilent Technologies chip), with 0.1% formic acid as the eluent. The sample was then fractionated on a C18 reverse-phase capillary column (75 µm×43 mm in the Agilent Technologies chip) at a flow rate of 200 nl/min, with a linear gradient of eluent B (0.1% formic acid in acetonitrile) in A (0.1% formic acid) from 5 to 60% in 50 min. Elution was monitored on the mass spectrometers without any splitting device. Peptide analysis was performed using data-dependent acquisition of one MS scan (m/z range from 400 to 2000 Da/e) followed by MS/MS scans of the three most abundant ions in each MS scan. Dynamic exclusion was used to acquire a more complete survey of the peptides by automatic recognition and temporary exclusion (2 min) of ions from which definitive mass spectral data had previously been acquired. Moreover a permanent exclusion list of the most frequent peptide contaminants (keratins and trypsin peptides) was included in the acquisition method in order to focus the analyses on significant data.

### Database Search

Mass spectral data obtained from both the MALDI MS and the LC-MS/MS analyses were used to search a non-redundant protein database using an in-house version of the Mascot (Matrix Science, Boston, MA, USA) software. The accurate peptide mass values from MALDI MS analyses were used in the Peptide Mass Fingerprint type of search taking into account the Carbamidomethyl-Cys as fixed modification, a peptide mass tolerance of ±100 ppm and a number of missed cleavages of 2. Peptide mass values and sequence information from LC-MS/MS experiments were used in the MS/MS Ion Search taking into account the Carbamidomethyl-Cys as fixed modification, a precursor ion and a fragment ion mass tolerance of ±600 ppm and 0.6 Da respectively. Only protein identifications with significant MASCOT score (p<0.05) were taken into consideration.

### Immuno-blot analysis

For the analysis of the platelet basic protein (PBP) and the Factor H (FH), 35 µg of total serum proteins were solubilised in sample buffer (50 mM Tris pH 6.8, 2% SDS, 10% glycerol, traces of bromophenol blue), under reducing (100 mM β-mercaptoethanol) or non reducing conditions, respectively. Samples were loaded on polyacrylamide gels, transferred to nitrocellulose membranes and probed with goat polyclonal anti-PBP antibody (clone T17, Santa Cruz Biotechnology, Santa Cruz, CA, USA) or mouse monoclonal anti-FH antibody (clone L20/3,Santa Cruz Biotechnology). Peroxidase conjugated sheep anti-mouse or donkey anti-goat IgG (GE Healthcare) were used as secondary antibodies and ECL-Plus (GE Healthcare) as chemiluminescence detection system.

### ELISA assay

FH in serum was quantified using the Human Complement Factor H ELISA kit (Hycult Biotech, Uden, The Netherlands) according to the manufacturer's protocol.

### Hemolytic assay

The hemolytic experiment was conducted as previously described [13]. 100 µl of serum were diluted in 2.5 mM barbital (Merck Chemicals, Nottingham, UK), 1.5 mM sodium barbital (Merck Chemicals), 144 mM NaCl (Sigma/Aldrich), 7 mM MgCl_2_ (Sigma/Aldrich), 10 mM EGTA (Sigma/Aldrich), pH 7.2–7.4. A duplicate of each sample was prepared in the same buffer plus 50 mM EDTA (Carlo Erba Reagenti, Milan, Italy) and was used as a blank. 200 µl of sheep erythrocytes (1×10^8^ cells/ml) were added to each sample and blank and the mixtures were incubated at 37°C under mixing. The reaction was stopped after 30 min with 2.5 mM barbital, 1.5 mM sodium barbital, 144 mM NaCl, 2 mM EDTA, pH 7.2–7.4. The mixtures were centrifuged and the hemolysis was determined by measuring the absorbance at 414 nm of the supernatants. The percentage of lysis of each sample was calculated by subtracting the A_414_ of the blank and dividing by the absorbance of the control of total lysis. Whenever indicated, 100 µl of each serum sample were incubated with 25 or 50 µg of Purified Human Factor H (Merck Chemicals Ltd., Nottingham, UK) to evaluate the effect of purified human FH on sheep red cell lysis.

### Factor H binding assay to endothelial cells

Human umbilical vein endothelial cells (HUVECs) were obtained according to the method of Jaffe et al. [14]. Cells were grown in a 12.5 cm^2^ culture flask filled with 10 ml of M-199 containing 10% fetal calf serum (FCS, Seromed, Berlin, Germany), 2 mM glutamine (Seromed, Berlin, Germany), 30 µg/ml endothelial cell growth supplement (Sigma-Aldrich, St. Louis, Mo, USA), 100 µg/ml heparin (Sigma-Aldrich, St. Louis, Mo, USA), 100 U/ml penicillin-streptomycin (Sigma-Aldrich, St. Louis, Mo, USA), 100 µg/ml streptomycin (Sigma-Aldrich, St. Louis, Mo, USA), and 2.5 µg/ml amphotericin (Sigma-Aldrich, St. Louis, Mo, USA). The flasks were incubated at 37°C, 100% humidity, and 5% of CO_2_. After reaching confluence, cells were sub-cultured in 75 cm^2^ culture flasks and medium was refreshed every 2 days.

Cells were used up to passage 4. Prior to harvesting, HUVECs were washed thoroughly in 2xPBS to remove FCS-derived FH and returned to serum-free media for 2 h before being detached by incubation in 0.01% EDTA/PBS. Cells were treated with patient and control sera (20% in 0.5xDPBS) for 20 min at 4°C and subsequently incubated with mouse monoclonal anti- human FH antibody (AbD Serotec) and Alexa-fluor 488–conjugated goat anti-mouse IgG (Invitrogen) 1∶100 at 4°C for 20 min. All washes and incubation steps were performed in 0.5xPBS containing 0.5% BSA. Control experiments were performed in the absence of serum.

Cells were examined by the fluorescence-activated cell sorter (FACScan, Becton-Dickinson Immunocytometry Systems, Mountain View, California, USA) equipped with Cell Quest software. Forward and sidewise scatters were used to define the fluorescent cell population and 10.000 events were routinely counted.

### Statistical analysis

Analysis of Variance was used to evaluate differences between controls and patients if concentrations of the identified proteins were normally distributed, according to the skewness-kurtosis test. The assumption of homoschedasticity was verified by Bartlett's test. Pair-wise comparisons were performed adjusting for multiple comparisons by Bonferroni correction if the global test was significant. The Kruskal-Wallis test was done for the functional analysis of serum FH.

## Results

### Clinical and demographic characteristics of SSc and ScGVHD patients

All SSc patients fulfilled the American College of Rheumatology criteria for the diagnosis of SSc [2] and were clustered into two groups: lSSc and dSSc subjects, according to the criteria of LeRoy et al [1]. In particular, patients with dSSc presented anti Scl70 Abs and patients with lSSc were positive for anti-centromere Abs, except patient 4 who presented only ANA (Table 1).

**Table 1:** Demographic and clinical data of SSc patients and healthy subjects.

Subject	Sex (M/F)	Age (yr)	Disease duration (yr)	Autoantibody profile	RSS	Lung fibrosis	Other clinical abnormalities	Current pharmacological treatment
Healthy								
1	F	28	NA	-	-	-	-	-
2	F	50	NA	-	-	-	-	-
3	M	50	NA	-	-	-	-	-
4	F	55	NA	-	-	-	-	-
5	F	68	NA	-	-	-	-	-
6	F	60	NA	-	-	-	-	-
7	F	545	NA	-	-	-	-	-
8	F	43	NA	-	-	-	-	-
9	M	43	NA	-	-	-	-	-
10	F	41	NA	-	-	-	-	-
11	F	63	NA	-	-	-	-	-
12	F	62	NA	-	-	-	-	-
13	M	33	NA	-	-	-	-	-
14	M	28	NA	-	-	-	-	-
15	F	43	NA	-	-	-	-	-
16	F	32	NA	-	-	-	-	-
lSSc								
1	F	75	5	ACA	3	no	no	Iloprost
2	F	36	5	ACA	7	no	no	Ilocrost
3	F	43	6	ACA	15	no	no	Iloprost
4	F	66	31	ANA	6	no	no	Iloprost
5	F	82	9	ACA	7	no	no	Iloprost
6	F	65	6	ACA	9	no	no	Iloprost
7	F	44	5	ACA	10	no	no	Iloprost
8	F	65	11	ACA	20	no	no	Iloprost
9	F	64	9	ACA	12	no	no	Iloprost
10	M	61	9	ACA	18	no	no	Iloprost
11	F	77	13	ACA	9	no	no	Iloprost
dSSc								
1	F	64	7	Scl-70	23	yes	no	Iloprost
2	F	30	8	Scl-70	35	yes	arrhythmia	Iloprost
3	F	74	9	Scl-70	7	yes	no	Iloprost
4	F	30	10	Scl-70	18	yes	no	Iloprost+ AZA
5	F	64	9	Scl-70	6	yes	no	Iloprost+MTX
6	F	68	13	Scl-70	14	no	arrhythmia	Iloprost
7	M	71	10	Scl-70	10	yes	arrhythmia	Iloprost
8	F	60	14	Scl-70	11	no	polmonary hypertension	Iloprost
9	F	64	9	Scl-70	11	yes	no	Iloprost+AZA
10	M	49	13	Scl-70	16	no	no	Iloprost
11	F	30	5	Scl-70	6	yes	no	Iloprost+AZA
12	F	39	6	Scl-70	13	no	no	Iloprost
13	F	66	6	Scl-70	7	yes	no	Iloprost+AZA
14	M	53	11	Scl-70	5	no	no	Iloprost
15	F	31	4	Scl-70	21	yes	no	Iloprost+AZA

lSSc: limited Systemic Sclerosis; dSSc: diffuse Systemic Sclerosis; F: female; M: male; ANA: anti-nuclear antibodies; ACA: anti-centromere antibodies; Scl-70: anti-topoisomerase I antibodies; mRSS: modified Rodnan Skin Score; MTX: methotrexate; AZA: azathioprine.

The skin involvement of SSc patients was quantified by the modified Rodnan Skin Score (mRSS) [15] and other organ abnormalities were evaluated with routinely examinations, including chest radiograph, electrocardiogram, colour-doppler echocardiogram and pulmonary function test. As shown in Table 1, no lSSc patients had important internal organ involvement whereas most of dSSc patients presented fibrosis alveolitis (*n* = 10), cardiac arrhythmia (*n* = 3) or pulmonary hypertension (*n* = 1) (Table 1). Conversely, none of the GVHD patients fulfilled the American College of Rheumatology criteria for the diagnosis of SSc. None of these patients presented anti-ENA antibodies, while ANA antibodies were found in six of them (Table 2). The measurement of skin involvement by the mRSS was not possible because ScGVHD patients presented cutaneous features in body areas that are not typical of SSc. In particular, sclerotic lesions were predominantly localized in the trunk and limbs and had been classified as morphea plaques or lichenoid eruptions. Only one patient presented diffuse induration of the skin. No internal organ involvement was detected in ScGVHD patients (Table 2).

**Table 2:** Demographic and clinical data of ScGVHD patients.

Subject	Sex (M/F)	Haematological disease	Age at BT (yr)	GVHD development after BMT (yr)	Autoantibody profile	Cutaneous features	Extracutaneous involvement	Current pharmacological treatment
ScGVHD								
1	M	ALL	23	3	ANA	Diffuse induration of skin	no	Iloprost
2	F	MDS	35	2	-	Lichen sclerosus	no	-
3	M	AML	34	5	ANA	Morphea	no	Iloprost
4	F	AML	57	3	ANA	Morphe	no	Iloprost
5	M	AML	53	8	ANA	Morphea	no	Iloprost
6	M	AML	31	6	ANA	Lichen sclerosus	no	-
7	F	AML	61	1	ANA	Morphea	no	Iloprost
8	M	AML	36	5	-	Morphea	no	Iloprost
T w/o GVHD								
1	M	AML	45	-	-	-	-	-
2	F	AML	53	-	-	-	-	-
3	M	ALL	27	-	-	-	-	-
4	M	AML	33	-	-	-	-	-
5	F	AML	32	-	-	-	-	-

ScGVHD: Sclerodermatous Graft-Versus-Host Disease; T w/o GVHD: bone marrow transplanted patients without Graft-Versus-Host Disease; F: female; M: male; ALL: acute lymphoblastic leukaemia; AML: acute myeloblastic leukaemia; MDS: myelodysplastic syndrome; BMT: bone marrow transplantation; BT: before transplantation; ANA: anti-nuclear antibodies.

Patients with haematological disorders underwent allogeneic haematopoietic stem cell transplantation, which was obtained from HLA-identical siblings. As conditioning regimen, they received cyclophosphamide associated with total body irradiation therapy. Then, they received methotrexate and cyclosporine for the GVHD prophylaxis (data not shown).

During this study, all SSc and ScGVHD patients received the cyclic Iloprost therapy, except for patients with lichenoid eruptions. Moreover, some dSSc patients with lung fibrosis (*n* = 6) were treated with immunosuppressive therapy (methotrexate or azathioprine).

### 2D serum maps of SSc patients differ from those of normal subjects

**Figure 1:**
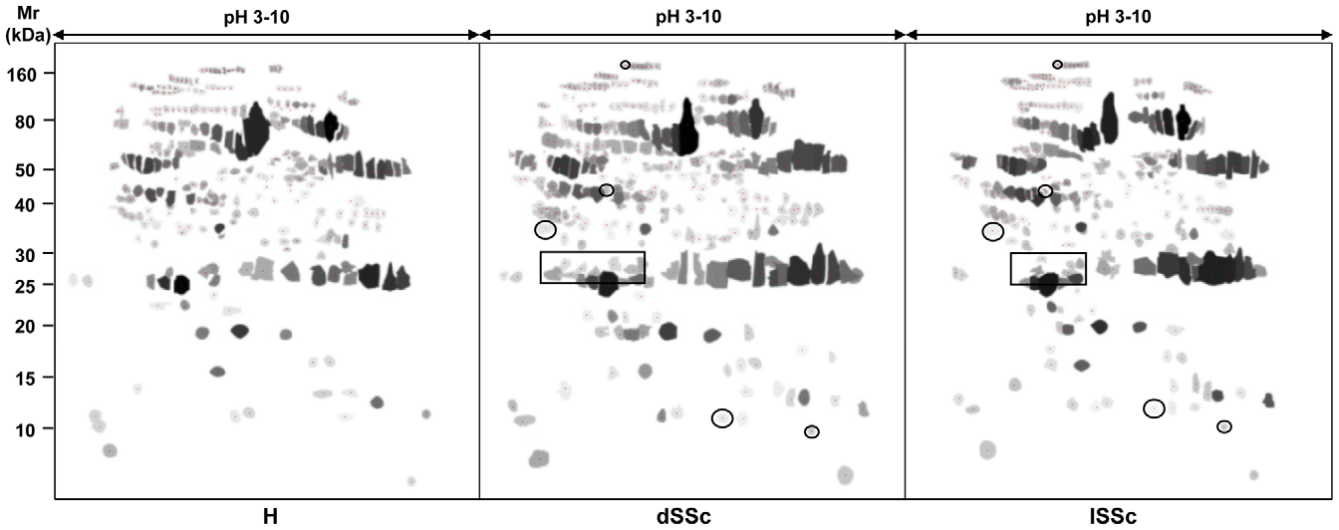
Synthetic referring gels of SSc patients and healthy controls. A comparative proteomic analysis of serum from SSc patients and healthy controls was conducted to identify differently expressed protein spots. Only protein spots that were present in almost 75% of the total samples of each subject category were included in the synthetic referring gels.

We generated pH 3–10 2D gels from serum of some SSc patients (lSSc *n* = 10; dSSc *n* = 10) and controls (healthy *n* = 5). To reduce the variability of each patient sample and to evaluate the data significance, we generated synthetic referring gels from pH 3–10 2D maps of each subject category by the GE 2D Image Master Platinum software analysis. Only protein spots that were present in almost 75% of the total samples of each subject category were included in the synthetic referring gels. For isolated spots included in the synthetic gel, the intensity, area and volume values of the original spot were assigned. The % volume and the % intensity values were recalculated according to the total volume and intensity of the new synthetic image. The disease referring gels were matched with the control one and protein spots that exhibited differences of more than 1.5 fold in intensity and volume between dSSc or lSSc and control, were selected for the identification (Fig. 1). Moreover, to better investigate the proteins present in the acidic gel region, we also generated pH 4–5.5 2D gels which were analysed as above described.

**Figure 2:**
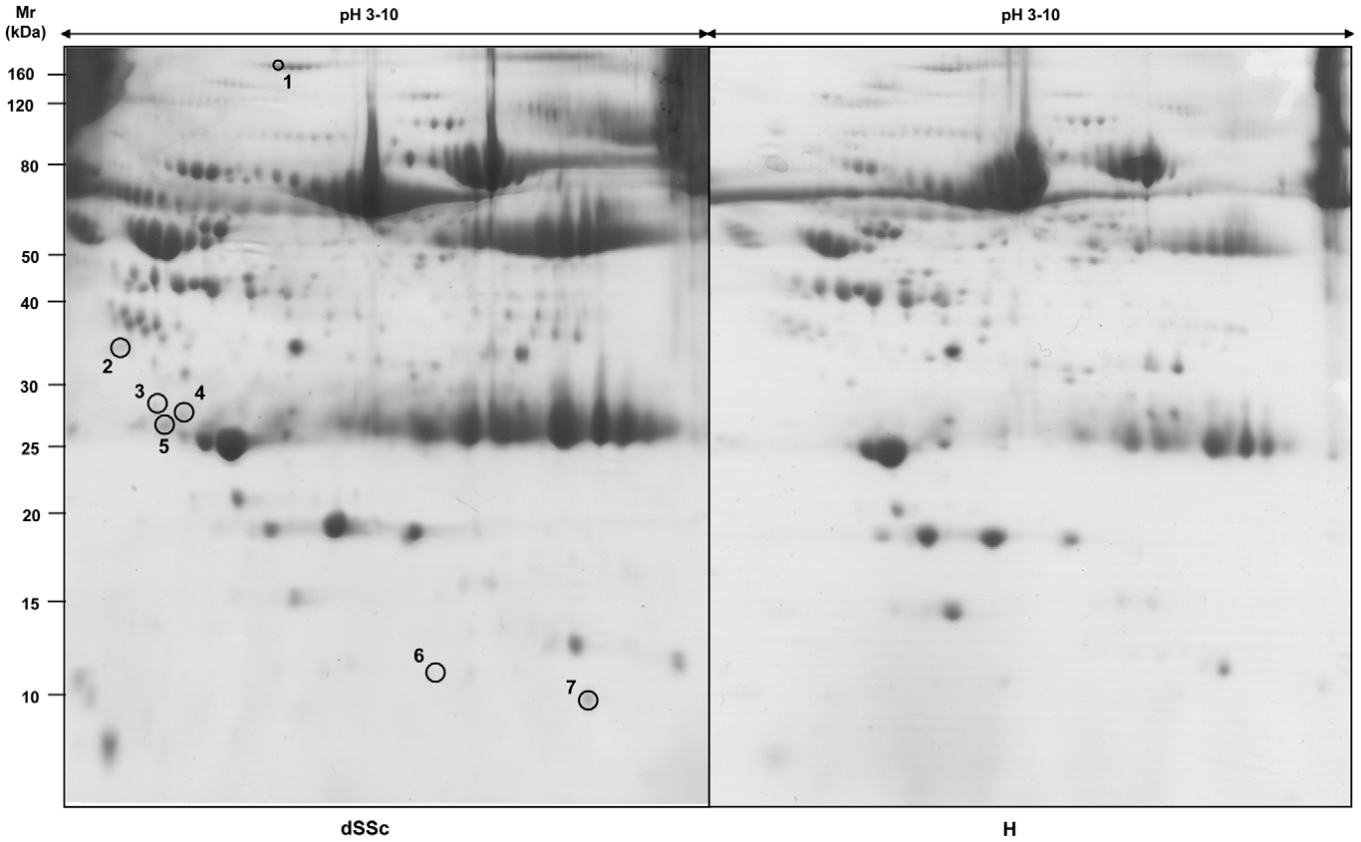
2D serum maps of a patient with diffuse SSc and a healthy control. 1200 µg of total serum proteins were focused on non-linear pH 3–10 immobiline dry strips and then separated into 9–16% polyacrilamide gels, which were stained with colloidal Coomassie. Marked are the protein spots, which have been identified by mass spectrometry.

**Figure 3:**
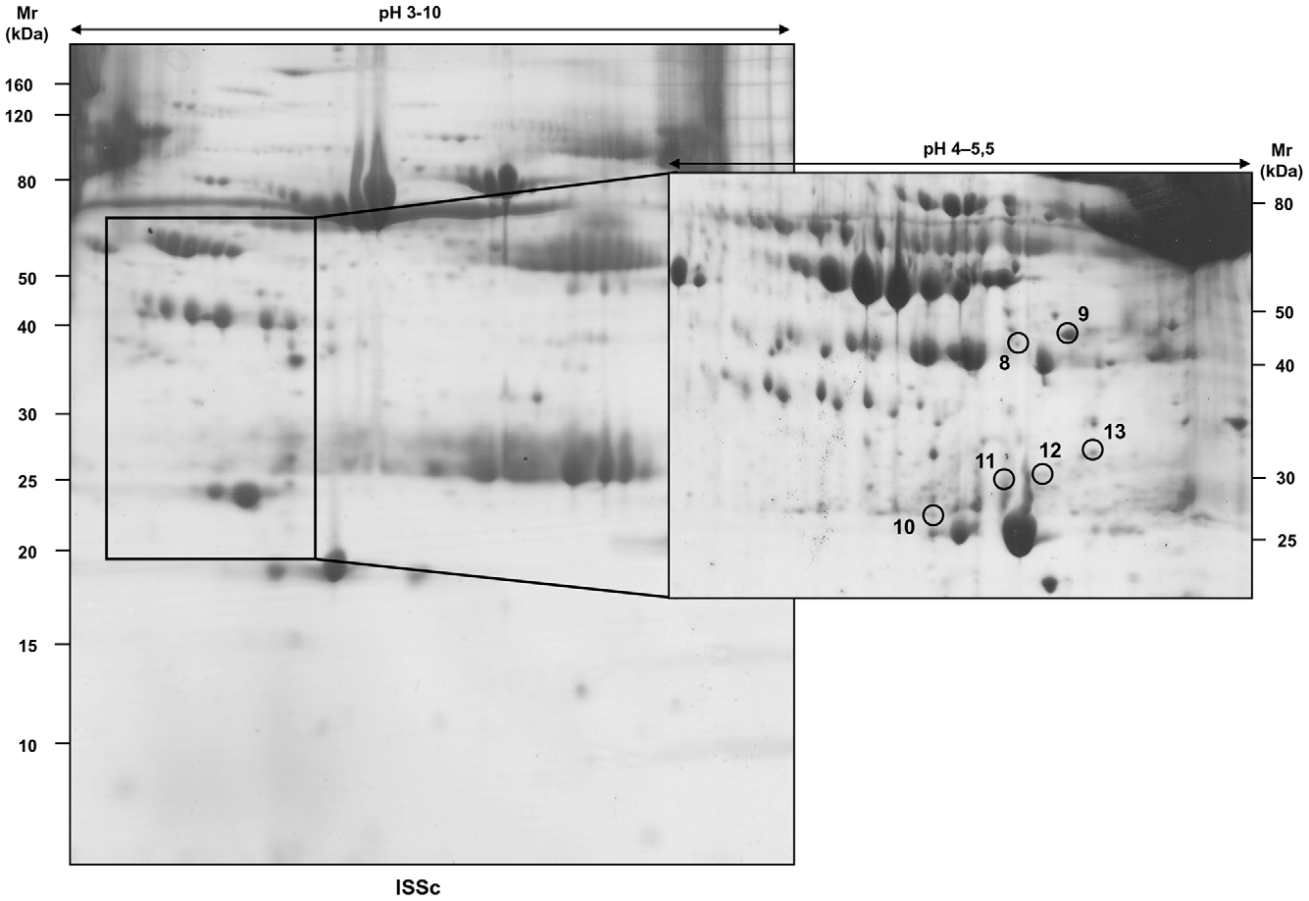
2D serum map of a patient with limited SSc. 1200 µg of total serum proteins were focused on non-linear pH 3–10 immobiline dry strips and then separated into 9–16% polyacrilamide gels, which were stained with colloidal Coomassie. Marked are the protein spots, which have been analysed by mass spectrometry.

This approach allowed us to highlight seven protein spots in pH 3–10 2D maps (Fig. 2) and six in pH 4–5.5 2D maps (Fig. 3), which were differently expressed in SSc patients, compared to healthy controls. These protein spots were excised from the gels and analysed by mass spectrometry (Table 3). We identified thirteen proteins, that were divided into five functional clusters: (i) cell protection, (ii) cell proliferation, (iii) acute phase response, (iv) immune response (v) host infection. Concentration differences of each identified protein had been expressed as ratio between the mean volume of the spot in each subgroup of patients and in healthy controls (Table 3).

**Table 3:** Biomarker candidates identified using proteomic analysis.

Spot nr.	Access.nr.	Protein name	Functional cluster	MS analysis	Coverage %	Disease vs control ratio *		
						dSSc	lSSc	ScGVHD
1	gi/85681919	Complement Factor H precursor	Cell protection	MALDI MS	7	1.75	1.90	1.63
2	gi/4507725	Transthyretin	Acute phase response	LC-MS/MS	39	n/d H	n/d H	n/d H
3	gi/229528	Protein Len, Bence Jones	Immune response	LC-MS/MS	22	n/d H	n/d H	n/d H
4	gi/229528	Protein Len, Bence Jones	Immune response	LC-MS/MS	22	n/d H	n/d H	n/d H
5	gi/4557321	Apolipoprotein A-I precursor	Acute phase response	LC-MS/MS	47	1.73	1.08	2.19
6	gi/225986	Amyloid related serum protein (SAA)	Cell proliferation Immune response	MALDI MS	75	1.16	1.51	2.03
7	gi/129874	Platelet basic protein precursor	Cell activation Immune response	MALDI MS	44	1.31	1.52	1.92
8	gi/93163358	Apolipoprotein A-IV precursor	Acute phase response	MALDI MS	34	4.74	9.44	14.24
9	gi/5174411	CD5 antigen-like	Immune response	MALDI MS	62	4.5	2.87	3.81
10	gi/693863	IgM autoantibody light chain, anti-GPIIb	Immune response	LC-MS/MS	15	n/d H	n/d H	n/d H
11	gi/1911815	Antitubulin IgG1 kappa VL chain	Immune response	LC-MS/MS	15	6.61	2.95	4.32
12	gi/15637439	Anti-pneumococcal capsular polysaccharide Ig light chain variable region	Infection	LC-MS/MS	13	n/d H	n/d H	n/d H
13	gi/4504489	Histidine-rich glycoprotein precursor	Not known	MALDI MS	21	0.30	0.54	0.13
14	gi/3603391	Anti Pneumococcal/anti dsDNA Ig L-chain Fab fragment	Infection	LC-MS/MS	30	n/d H	n/d H	n/d H

MS: mass spectrometry; n/d H: not detected in healthy subjects.

*: Disease vs control ratio is calculated as the mean volume in the diseased subjects divided by the mean volume in control subjects.

Some of the identified proteins such as the amyloid related serum protein (SSA) and the apolipoprotein A–I (apoA-I) had already been described as being involved in SSc [16], [17], whereas the Factor H (FH) and platelet basic protein (PBP) were firstly identified in the present study.

We then asked whether patients with ScGVHD may present similarities with the natural occurring SSc form.

### Serum protein expression profile of ScGVHD patients is similar to that of SSc patients, except for anti-Pneumococcal/anti-double-stranded DNA antibodies

**Figure 4:**
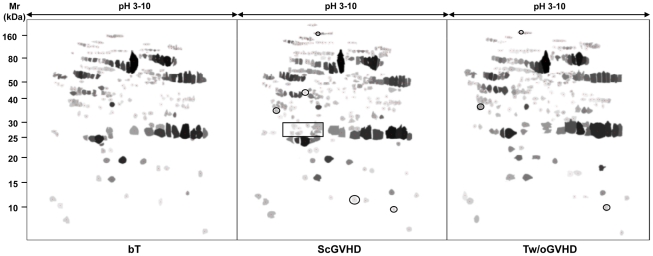
Synthetic referring gels of haematological patients. A comparative proteomic analysis of serum from patients with ScGVHD or without ScGVHD and before BMT was conducted to identify differently expressed protein spots. Only protein spots that were present in almost 75% of the total samples of each subject category were included in the synthetic referring gels.

The comparative proteomic analysis was conducted on serum of some SSc and ScGVHD patients (lSSc *n* = 10; dSSc *n* = 10; ScGVHD *n* = 8). In addition, we evaluated 2D serum maps of T patients without GVHD (*n* = 5) and ScGVHD patients before BMT (*n* = 8). To simplify the image master 2D software analysis, we created synthetic referring gels of each haematological patient subgroups, as previously described (Fig. 4). The serum protein profile of ScGVHD patients resulted quite similar to that of SSc patients (Fig. 1, Table 3), but was different from those of ScGVHD patients before BMT and T patients without GVHD (Fig. 4).

**Figure 5:**
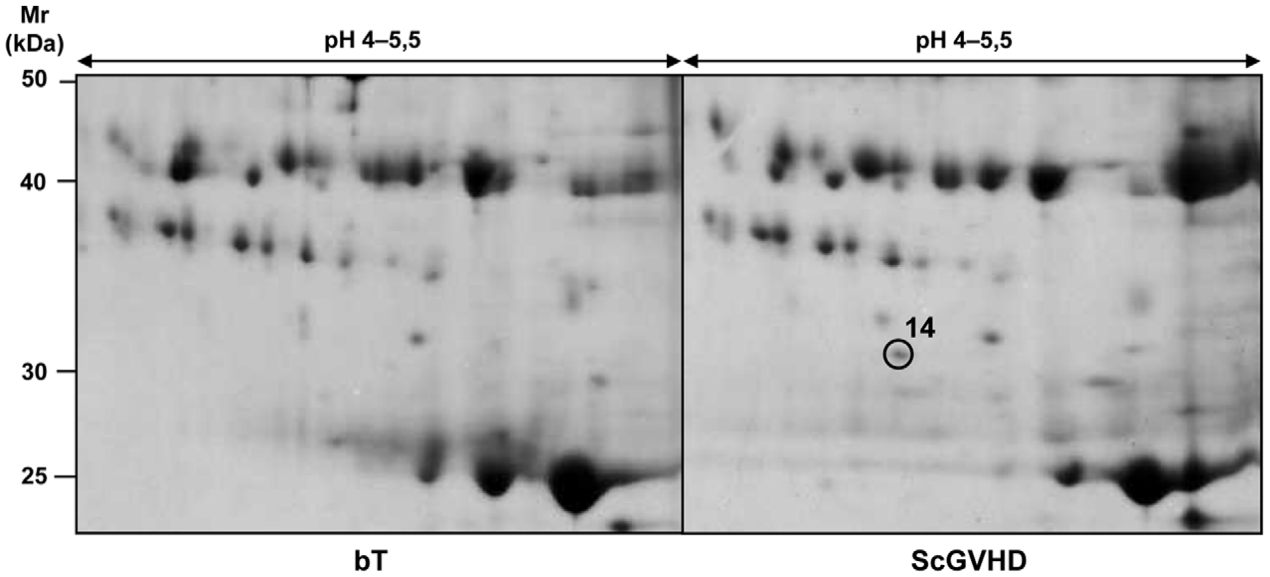
2D serum maps of a patient before BMT and after ScGVHD. 1200 µg of total serum proteins were focused on pH 4–5.5 immobiline dry strips and then separated into a 9–16% polyacrilamide gels; gels were stained with colloidal Coomassie. Marked is the protein spot, which has been analysed by mass spectrometry.

It is interesting to note that we found anti-Pneumococcal/anti-double-stranded DNA antibody fragments only in serum of T patients with ScGVHD, not in serum of the same subjects before BMT (Fig. 5) and not even in serum of T patients without ScGVHD or in SSc subjects, except for patient 4 with lSSc, and healthy controls.

### Factor H is increased and modulated in dSSc and ScGVHD patients

We investigated two proteins out of those identified by comparative proteomic analysis that might be new possible functional biomarkers involved in the pathogenesis of SSc and ScGVHD disorders: PBP, which contributes to the extracellular fibrosis and FH which is essential for the vascular EC protection from the complement lysis [18], [19].

**Figure 6:**
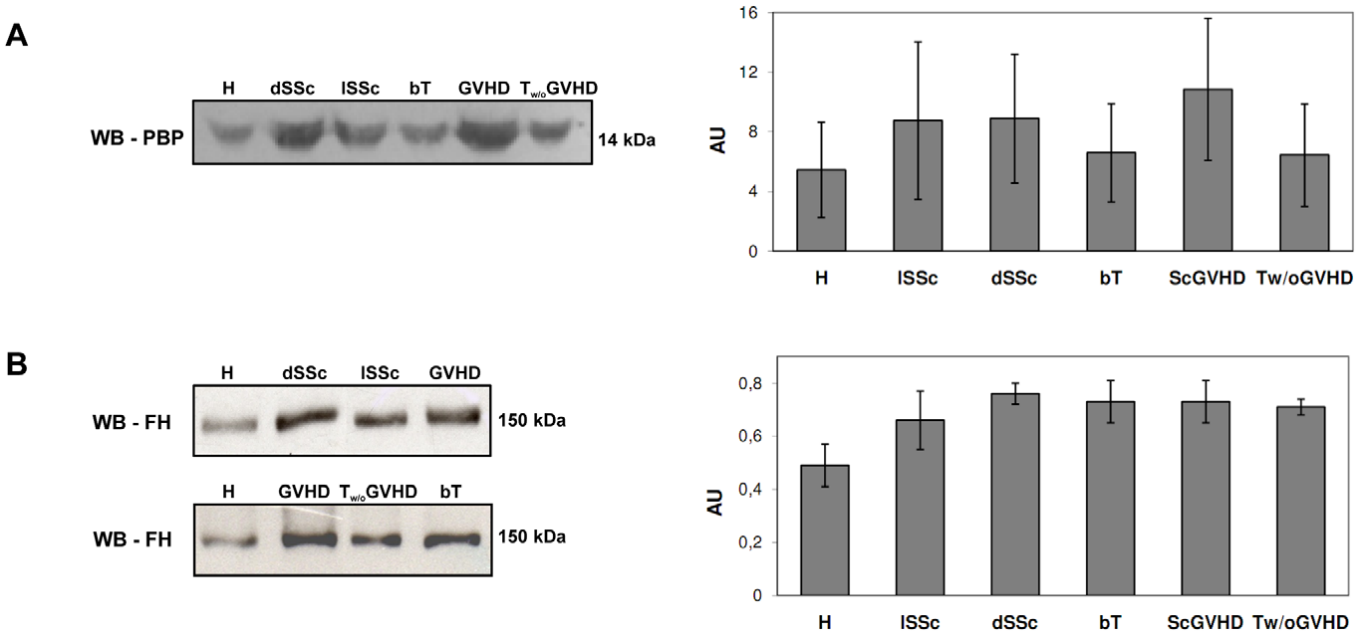
Immuno-blot analysis of complement FH and PBP. (**A**) Immuno-blot analysis of PBP and graph reporting the quantification of PBP derived protein expression as detected by densitometric analysis. 35 µg of total serum proteins were solubilised under reducing conditions, loaded on 12% polyacrylamide gels, transferred to nitrocellulose membranes and probed with goat polyclonal anti-PBP antibody. We could not separate by this technique the individual peptides of this family because of their small differences in the amino acid sequence length. Shown is one representative experiment of five with similar results. (**B**) Immuno-blot analysis of FH and graph reporting the quantification of FH expression as detected by densitometric analysis. FH molecules from serum of dSSc, lSSc, ScGVHD patients and H, bT and T w/o GVHD subjects were separated by mono-dimensional electrophoresis on 8% polyacrylamide gels under non reducing conditions. Samples were transferred to nitrocellulose membranes and probed with mouse monoclonal anti-FH antibody. Shown is one representative experiment of five with similar results.

We carried out immuno-blot analysis with specific antibodies against FH and PBP from serum of five subjects of each patient and control subgroup. Serum levels of PBP derived peptides were similar in SSc and ScGVHD patients compared to controls (Fig. 6 A) and the global test was not significant (p = 0.2292). A similar level of PBP derived peptides was also found between SSc and ScGVHD patients (Mean Difference: -2.02. C.I.95%: -6.95; 2.91).

As shown in Fig. 6 B, FH serum levels were significantly increased in dSSc patients (p = 0.003) and almost reached significance in ScGVHD patients (p = 0.009) compared to healthy controls, as also confirmed by Bonferroni-adjusted pairwise comparisons after Analysis of Variance (Table 4). A similar concentration of FH was found between SSc and ScGVHD patients (Mean Difference: -0.02. C.I.95%: -0.14; 0.11). High levels of FH were also observed in T patients who did not develop ScGVHD (Fig. 6 B).

**Table 4:** Differences in FH concentration, using immuno-blotting.

Factor H (Western Blotting)			
Global Test	Multiple Comparisons		
P = 0.0024	Sample A	Sample B	p-value*
	Healthy	lSSc	0.084
Healthy (n = 5; median: 0.52; Q1-Q3: 0.43-0.56)	Healthy	dSSc	0.003
dSSc (n = 5; median: 0.77; Q1-Q3: 0.74-0.79)	Healthy	ScGVHD	0.009
ScGVHD (n = 5; median: 0.76; Q1-Q3: 0.69-0.77)	lSSc	dSSc	0.525
lSSc (n = 5; median: 0.68; Q1-Q3: 0.59-0.72)	lSSc	ScGVHD	1.000
	dSSc	ScGVHD	1.000

The Kruskal-Wallis test was used for the statistical analysis of the data.

*A p-value less than 0.0083 was considered significant, according to Bonferroni correction.

Then, we performed ELISA assay for FH analysis in a larger cohort of SSc and healthy subjects (lSSc *n* = 11; dSSc *n* = 13; healthy *n* = 9; ScGVHD *n* = 4) that confirmed what we documented with immuno-blot analysis (Table 5).

**Table 5:** Differences in FH concentration, using ELISA assay.

Factor H (ELISA assay)			
Global Test	Multiple Comparisons		
P = 0.00114	Sample A	Sample B	p-value*
	Healthy	lSSc	0.015497
Healthy (n = 9; median: 904.7067; Q1-Q3: 903.316-944.5305)	Healthy	dSSc	0.022007
dSSc (n = 13; median: 1192.891; Q1-Q3: 971.8707-1369.209)	Healthy	ScGVHD	0.000038
ScGVHD (n = 4; median: 1753.066; Q1-Q3: 1720.969-1985.02)	lSSc	dSSc	0.407116
lSSc (n = 11; median: 1061.01; Q1-Q3: 961.6105-1391.766)	lSSc	ScGVHD	0.007990
	dSSc	ScGVHD	0.004286

The Kruskal-Wallis test was used for the statistical analysis of the data.

*A p-value less than 0.004167 was considered significant, according to Bonferroni correction.

We then performed a functional analysis using serum from patients (lSSc *n* = 11; dSSc *n* = 15; ScGVHD *n* = 7) and controls (healthy *n* = 15) to evaluate the ability of FH to control complement activation on cell surfaces. The hemolytic assay was conducted using sheep erythrocytes that resist to human complement-mediated hemolysis but are more sensitive than human red cells when a perturbation of FH function is present [20]–[22]. According to the Bonferroni-adjusted pairwise comparisons after Kruskal-Wallis test, we observed significant increased hemolysis with serum from dSSc patients, compared with serum of either lSSc patients (Table 6, dSSc *vs* lSSc: p<0.001) or ScGVHD patients (dSSc *vs* ScGVHD: p<0.001), but no significant differences were observed compared to healthy controls (dSSc *vs* H: p = 0.031), since the threshold for significance was set to p = 0.004167 and not to p = 0.05, due to pairwise correction.

**Table 6:** Sheep erythrocyte lysis by human sera.

Lysis (%)			
Global Test	Multiple Comparisons		
P = 0.00064	Sample A	Sample B	p-value*
	Healthy	lSSc	0.025921
Healthy (n = 15; median: 3.63; Q1-Q3: 2.18-5.83)	Healthy	dSSc	0.031100
dSSc (n = 15; median: 10.72; Q1-Q3: 3.00-52.66)	Healthy	ScGVHD	0.050120
ScGVHD (n = 7; median: 1.87; Q1-Q3: 0.12-3.68)	lSSc	dSSc	0.000126
lSSc (n = 11; median: 1.19; Q1-Q3: 0.28-2.51)	lSSc	ScGVHD	0.483931
	dSSc	ScGVHD	0.000870

The Kruskal-Wallis test was used for the statistical analysis of the data.

*A p-value less than 0.004167 was considered significant, according to Bonferroni correction.

**Figure 7:**
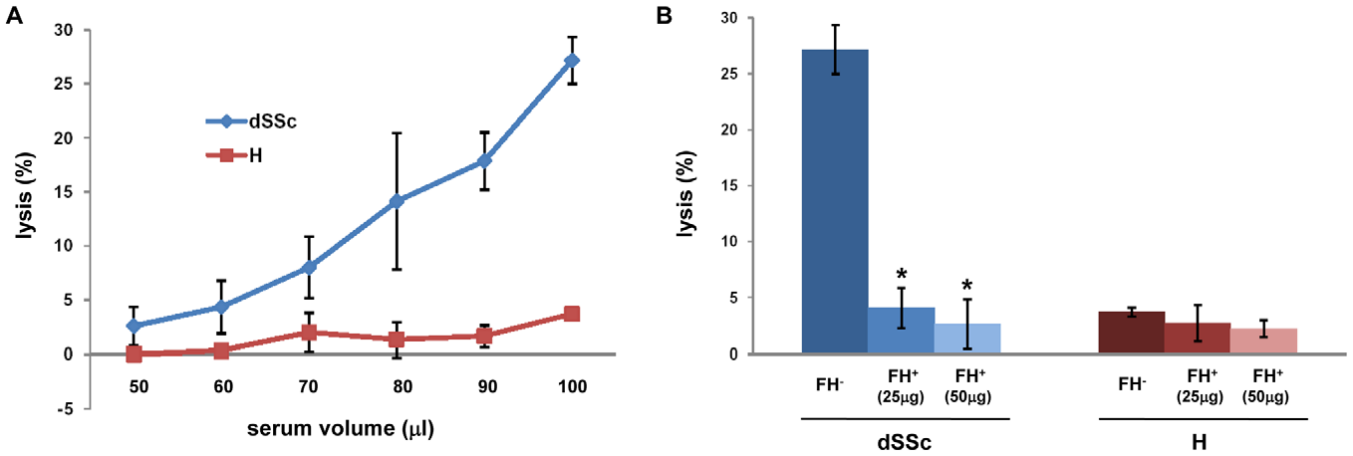
Hemolysis of sheep erythrocytes. (**A**) Lysis of sheep erythrocytes by human serum. 50-100 µl of serum from dSSc patients (*n* = 3) and healthy controls (*n* = 3) were incubated with 200 µl of sheep erythrocytes (1×10^8^ cells/ml). Hemolysis was determined by measuring the absorbance at 414 nm of cell supernatants. Data are presented as percentage of the total lysis. (**B**) Addition of purified FH on sheep erythrocytes. Sheep erythrocytes (1×10^8^ cells/ml) were preincubated with 100 µl of serum from dSSc and H patients and then incubated with 25 or 50 µl of human purified FH. Data are presented as percentage of the total lysis.

We also carried out the hemolysis assay either with increasing amounts of dSSc serum or in presence of exogenous purified human FH. As shown Fig. 7 A, increasing amounts of dSSc serum induced a higher degree of haemolysis compared to serum from normal controls. The addition of purified human FH to serum of dSSc patients significantly prevented the complement-mediated hemolysis in serum from dSSc patients (Fig. 7 B).

**Figure 8:**
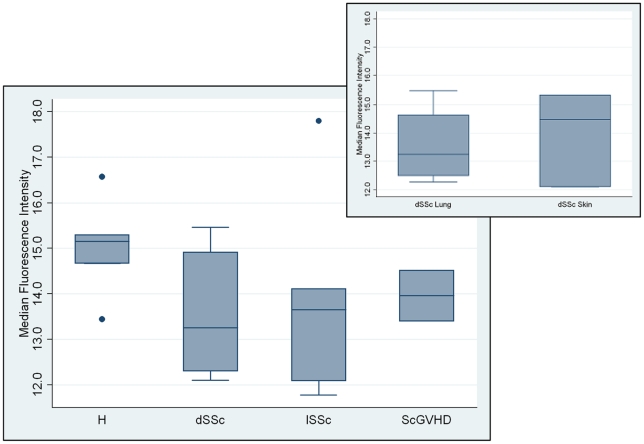
Factor H binding to endothelial cells. HUVEC were incubated in patient and control sera (dSSc *n* = 8; lSSc *n* = 6; ScGVHD *n* = 3; healthy *n* = 5) and bound FH was visualized with a specific antibody by flow cytometry. The results obtained were compared using box and whiskers plot. **Figure inset.** Two subtypes of dSSc patients are represented: patients with important pulmonary involvement (dSSc lung, *n* = 4) and patients with exclusive cutaneous involvement (dSSc skin, *n* = 4). The results obtained were compared using box and whiskers plot.

To validate these results, the *in vitro* binding of FH to human ECs was investigated with HUVECs, which had been used as model of self-cells. We incubated HUVECs with human sera from patients and healthy subjects (lSSc *n* = 6; dSSc *n* = 8; ScGVHD *n* = 3; healthy *n* = 5) and the binding of native FH was measured by flow cytometry. We observed reduced surface-bound FH when HUVECs were incubated with the serum from dSSc patients (Fig. 8), in particular in dSSc patients with pulmonary involvement (Figure inset). For the other cases, the large variability in FH cell binding documented for lSSc patients and the limited number of ScGVHD patients that we analysed, did not allow us to formulate a conclusion.

## Discussion

Here, we used the comparative proteomic approach to identify new differently expressed proteins in patients with SSc compared with healthy subjects and to evaluate, for the first time, a population of T patients who developed sclerotic lesions related to GVHD.

In agreement with previous studies on SSc patients, we observed high levels of amyloid related serum protein (SSA) in serum of SSc patients [23], but we firstly found that SSA was also increased in ScGVHD patients. Since SSA has been described as being involved in chemotaxis of inflammatory cells, modulation of proinflammatory cytokines and endothelial cell proliferation [24]–[28], the present data support a possible role of SSA in the development of ScGVHD.

In addition, we found that CD5 antigen-like molecule and apoA-I were increased in SSc and ScGVHD patients compared to healthy controls. The CD5 antigen-like molecule seems to play a critical role in autoimmune disorders, mediating B lymphocytes [29], [30], whereas the apoA-I improves vascular complications in a mouse model of SSc [17].

We also identified another protein, PBP, possibly important in the development of fibrosis in SSc patients, based on the observation that PBP regulates human fibroblast growth by activation of platelet-derived growth factor receptor [31]–[33], whereas its derivate, the connective tissue-activating peptide-III (CTAP-III), stimulates the glycosaminoglycan formation, participating in the extracellular matrix remodelling. Although we did not find significant changes in PBP serum levels of disease subjects compared to controls, the acid form of PBP suggests that the PBP mobility shift in 2D maps might be related to PBP post-translational modifications, e.g. protein phosphorylation state, more than to PBP quantitative differences (Table 3, Table S1) [34]. Others proteins show differences on experimental isoelectric point (pI) and/or molecular weight (MW) compared with their theoretical values in 2D maps of diseased subjects, suggesting possible post-translational modifications (Table 3, Table S1). We observed an acidic isoform of Transthyretin (spot 2) most likely related to a protonation of one of its Lysine residue [35]; the presence of Bence Jones proteins with lower pI compared to the theorical one (spots 3 and spot 4) might also suggest protonation of one or more Lysine/Arginine residues; the Histidine-rich glycoprotein precursor (spot 13) with lower experimental MW compared to the theoretical one, possibly caused by deglycosylation of the protein.

FH is another novel protein that we found differently expressed in both SSc and ScGVHD patients compared to normal controls and has been never reported before. We observed that FH (spot 1) was placed in disease 2D gels at higher MW than the theoretical one, which might be related to a saccharide portion that remains still complexed to Asparagine at the C-terminal of the protein (Table 3, Table S1) [36], [37]. FH is an important regulator of the alternative complement pathway, which can be activated by a variety of polysaccharides, bacteria and viruses [38], [39]. The consequence of complement activation *via* the alternative pathway is the indiscriminate C3b binding to self and foreign cellular surfaces, resulting in cell lysis. FH protects host cells from complement mediated damage by binding ECs and inactivating the C3b fragment [40], [41].

Elevated serum FH levels, which we found in all the subgroups of patients, may be related to the increased FH cellular release for self protection against complement attack during an infection or inflammatory disease [42], [43]. A previous study has shown that acute myeloid leukemia blasts produce factors increasing the complement protein synthesis by human hepatocytes *in vitro* [44] but so far no data are available on serum FH levels in onco-haematological patients after chemotherapy or radiation therapy. These data, together with the identification of anti-Pneumococcal/anti double-stranded DNA antibodies in serum of all patients with ScGVHD, which should be further validated with more specific techniques, open an interesting scenario on the possible role of infectious agents and regulator complement proteins in the etio-pathogenesis of this disease [45]. In fact, there are evidence about the pivotal role of FH in mediating the pneumococcus adhesion to human ECs in *vitro* [46], [47].

**Figure 9:**
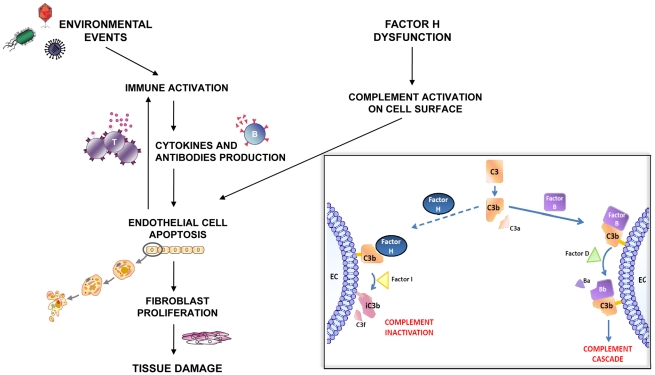
Schematic diagram of the working hypothesis. Increasing evidence suggests that damage to the vascular endothelium may be an early event in the SSc disease. It has been already shown that environmental agents and immune mechanisms may be the possible effectors of such an insult, but the cause of the higher host susceptibility to them remains unclear. We propose that endothelium of patients with dSSc may be not adequately protected because of a defective defensive barrier on cellular surface. FH is an important complement regulator, which protects host cells from complement mediated damage by binding ECs and inactivating the C3b fragment (**Figure inset**). A defective FH binding to ECs could contribute to the vascular damage, not providing host cells with protection against complement attack during an inflammatory insult.

However, the reduced FH binding to HUVECs and the increased lysis of sheep red cells after addition of serum from dSSc patients suggest a perturbation of the attachment of the soluble FH to the cellular surface at least in this subgroup of patients, as supported by the fact that increased lysis of sheep red cell was prevented by the addition of exogenous FH. In this case, we propose that dSSc patients may have a defect of the defensive barrier on cellular surface that could predispose them to endothelial damage. FH dysfunction might promote complement-mediated cellular damage and expose intracellular antigens, which cross-react with anti-bacterial antigen antibodies, amplifying and perpetuating the activity of the immune system against the host (Fig. 9). An aberrant expression of complement components on EC surface and high plasma levels of cleavage products of activated alternative pathway have already been detected in the early active phase of dSSc patients [48], [49]. Moreover, it has been shown in an animal model of autoimmune encephalomyelitis that purified human FH administration reduces both clinical score and tissue inflammation [50].

In conclusion, we believe that this study provides new elements which could be involved in EC damage with possible clinical implications for SSc and ScGVHD patients. Further studies need to be carried out to evaluate the effective pathological role of these peptides in the above diseases.

## Supporting Information

Table S1 Biomarker candidates identified using proteomic analysis.
(0.05 MB DOC)
