# Supplementary material for: Comparative Proteomic Analysis of Serum from Patients with Systemic Sclerosis and Sclerodermatous GVHD. Evidence of Defective Function of Factor H
Source: PLoS One. 2010 Aug 13;5(8):e12162. doi: 10.1371/journal.pone.0012162 (PMC2921355; doi:10.1371/journal.pone.0012162)
Supplement: Table S1 — Biomarker candidates identified using proteomic analysis. (0.05 MB DOC) [file pone.0012162.s001.doc]

**Table S1.** Biomarker candidates identified using proteomic analysis

| Spot nr. | Access.nr. | Protein name | Functional cluster | MS analysis | Theoretical  MW/pI | Nr of  identified  peptides | Coverage  % | Mascot  score | Disease vs control ratio * | | |
| --- | --- | --- | --- | --- | --- | --- | --- | --- | --- | --- | --- |
|  |  |  |  |  |  |  |  |  | dSSc | lSSc | ScGVHD |
| 1 | gi/85681919 | Complement factor H precursor | Cell protection | MALDI MS | 165000 /5,4 | 7 | 7 | 59 | 1.75 | 1.90 | 1.63 |
| 2 | gi/4507725 | Transthyretin | Acute phase response | LC-MS/MS | 35400/5,52 | 5 | 39 | 239 | n/d H | n/d H | n/d H |
| 3 | gi/229528 | Protein Len, Bence Jones | Immune response | LC-MS/MS | 24499/9,22 | 3 | 22 | 168 | n/d H | n/d H | n/d H |
| 4 | gi/229528 | Protein Len, Bence Jones | Immune response | LC-MS/MS | 24499/9,22 | 4 | 22 | 184 | n/d H | n/d H | n/d H |
| 5 | gi/4557321 | Apolipoprotein A-I precursor | Acute phase response | LC-MS/MS | 28944/5,27 | 11 | 47 | 478 | 1.73 | 1.08 | 2.19 |
| 6 | gi/225986 | Amyloid related serum protein (SAA) | Cell proliferation Immune response | MALDI MS | 11675/5,89 | 5 | 75 | 139 | 1.16 | 1.51 | 2.03 |
| 7 | gi/129874 | Platelet basic protein precursor | Cell activation Immune response | MALDI MS | 10200/8,95 | 8 | 44 | 90 | 1.31 | 1.52 | 1.92 |
| 8 | gi/93163358 | Apolipoprotein A-IV precursor | Acute phase response | MALDI MS | 43358/5,18 | 16 | 34 | 202 | 4.74 | 9.44 | 14.24 |
| 9 | gi/5174411 | CD5 antigen-like | Immune response | MALDI MS | 38000/5,28 | 22 | 62 | 262 | 4.5 | 2.87 | 3.81 |
| 10 | gi/693863 | IgM autoantibody light chain, anti-GPIIb | Immune response | LC-MS/MS | 12686/9,51 | 1 | 15 | 60 | n/d H | n/d H | n/d H |
| 11 | gi/1911815 | Antitubulin IgG1 kappa VL chain | Immune response | LC-MS/MS | 24028/6,37 | 2 | 15 | 98 | 6.61 | 2.95 | 4.32 |
| 12 | gi/15637439 | Anti-pneumococcal capsular polysaccharide Ig light chain variable region | Infection | LC-MS/MS | 10584/8,75 | 1 | 13 | 67 | n/d H | n/d H | n/d H |
| 13 | gi/4504489 | Histidine-rich glycoprotein precursor | Not known | MALDI MS | 57660/7,03 | 11 | 21 | 136 | 0.30 | 0.54 | 0.13 |
| 14 | gi/3603391 | Anti Pneumococcal/anti dsDNA Ig L-chain Fab fragment | Infection | LC-MS/MS | 11303/9,26 | 2 | 30 | 72 | n/d H | n/d H | n/d H |

MS: mass spectrometry; MW: molecular weight; pI: isoelectric point; n/d H: not detected in healthy subjects

*: Disease vs control ratio is calculated as the mean volume in the diseased subjects divided by the mean volume in control subjects.
